# Bronchodilator Response Assessment of the Small Airways Obstructive Pattern

**DOI:** 10.2174/1874306401711010047

**Published:** 2017-07-25

**Authors:** Plamen Bokov, Clémence Martin, Sémia Graba, Karine Gillet-Juvin, Mohamed Essalhi, Christophe Delclaux

**Affiliations:** 1 AP-HP ; Hôpital Européen Georges Pompidou ; Service de Physiologie, Paris. France; 2 Université Paris Descartes, Sorbonne Paris Cité, Faculté de Médecine, Paris. France

**Keywords:** Pulmonary function testing, Bronchodilator response, Impulse oscillometry, Obstructive defect, Inspiratory capacity, Distal airways

## Abstract

**Background:**

A concomitant decrease in FEV_1_ and FVC with normal FEV_1_/FVC ratio and TLC defines small airways obstructive pattern (SAOP) and constitutes a classic pitfall of pulmonary-function-tests interpretation.

**Objective:**

To evaluate the prevalence of flow- (FEV_1_ increase≥12% and 200 mL), volume- (FVC or inspiratory capacity [IC] increase≥12% and 200 mL), flow and volume-, and non-response to bronchodilation in patients with SAOP. An additional objective was to assess whether impulse oscillometry (IOS) parameters allow the diagnosis of SAOP and its reversibility.

**Methods:**

Fifty consecutive adult patients with SAOP (FEV_1_ and FVC < lower limit of normal, FEV_1_/FVC and TLC > lower limit of normal) diagnosed on spirometry and plethysmography underwent the assessment of reversibility (400 µg salbutamol) on FEV_1_, FVC, IC and IOS parameters.

**Results:**

The diseases most frequently associated with SAOP were COPD and asthma (26 and 15 patients, respectively). Six patients were flow-responders, 20 were volume-responders, 9 were flow and volume-responders and 15 patients were non-responders. Overall, 26 patients had a significant improvement of IC, and 35 / 50 (70%, 95%CI: 57-83) exhibited a significant bronchodilator response. The difference between Rrs_5Hz_ and Rrs_20Hz_ was increased in 28/50 patients (56%, 95%CI: 42-70 with value higher than upper limit of normal) and its decrease after bronchodilator significantly correlated to FEV_1_ increase only, suggesting proximal airway assessment.

**Conclusion:**

A significant reversibility, mainly assessed on IC increase, is frequent in Small Airways Obstructive Pattern. Impulse oscillometry is of limited value in this context because of its low sensitivity.

## 1 INTRODUCTION

Pellegrino *et al.* in the interpretative strategies for lung function tests stated that special attention must be paid when FEV_1_ and FVC are concomitantly decreased and the FEV_1_/FVC ratio is normal or almost normal. Apart incomplete inhalation or exhalation, these authors stated that a “possible cause of this pattern is patchy collapse of small airways early in exhalation” [1]. This small airways obstructive pattern (SAOP) is not infrequent, represents ~ 7% to 10% of all pulmonary function tests in two large databases and is not specific of any disease in adults [2, 3]. It can also be encountered in asthmatic children [4]. Since this pattern is deemed to be the consequence of “patchy collapse of small airways early in exhalation” one may wonder whether bronchodilator response should be assessed on flow (increase in FEV_1_), volume (FVC and/or inspiratory capacity [IC] [5]) or both flow and volume. Moreover, improvements in IC correlate with improvements in exercise tolerance and endurance, which are recognized as important goals of disease management. Consequently, the primary objective of our study was to assess whether bronchodilator response should be assessed conventionally using FEV_1_ and FVC, or using IC.

SAOP has initially been defined as a pseudo-restrictive syndrome because of the reduction of FEV_1_ and FVC with normal FEV_1_/FVC ratio. If TLC measurement is made using dilution techniques, one may wrongly conclude that the defect is restrictive [3]. Since body plethysmography is not available in all pulmonary function testing laboratories, another method for SAOP detection is mandatory. Impulse oscillometry (IOS) has the advantage of being simple to use and is effort-independent [6]. The contribution of the distal airways could be determined by the fall in resistance from 5 Hz to 20 Hz (ΔRrs_5Hz-20Hz_) [6] and could therefore be useful to detect SAOP. Our additional objective was to assess the usefulness of IOS to diagnose SAOP (sensitivity) and its reversibility.

## 2 MATERIAL AND METHODS

### 2.1 Patients

We enrolled prospectively 50 consecutive Caucasian patients exhibiting SAOP defined by a decrease in both FEV_1_ and FVC (< lower limit of normal [LLN]) and normal (above LLN) FEV_1_/FVC ratio and TLC (the latter being measured by body plethysmography), while receiving no bronchodilator treatment. Lung transplantation was a non inclusion criterion.

The study was approved by the Institutional Review Board of the French learned society for respiratory medicine -SPLF- (CEPRO-2016-014) and all patients gave informed consent.

### 2.2 Pulmonary Function Tests

Complete lung function testing was obtained with the MasterScreen™ PFT system (Master Scope Body, MS-IOS, Jaeger, Carefusion Technologies, Yorba Linda, California, USA), according to international recommendations [7, 8].

IOS parameters: Pressure oscillation with frequencies varying between 5 and 35 Hz were superimposed on tidal breathing, producing recordable waveforms. Advanced signal processing of the impedance of the respiratory system (Zrs) was then used to extract the respiratory mechanic components from the recorded waveforms. The primary outcomes resistance of the respiratory system (Rrs) and reactance (Xrs) were plotted against frequency. Resistance is defined as the ratio of the pressure drop (kPa) over an airway segment and the flow (L·s^−1^) through that segment. Reactance is simplistically described as the amount of recoil generated against the pressure wave. We used the following IOS variables: impedance, resistance and reactance at 5 Hz; the fall in resistance between R5 and R20 (ΔRrs_5Hz-20Hz_), reflecting “peripheral” airway resistance; and the square root of the integrated area of low frequency reactance (AX), assumed to reflect the reactance of the “peripheral” airways, and serving as a confirmatory index to ΔRrs_5Hz-20Hz_.

At baseline were performed IOS, spirometry, slow vital capacity and static volumes measurements. Bronchodilator response to 400 µg salbutamol was evaluated using spirometry, slow vital capacity (IC recording) and IOS measurements. Significant bronchodilator response was defined according to recommendations for FEV_1_ and FVC [1], and by an increase in IC of at least 12% from baseline and ≥ 200 mL as proposed by Celli and colleagues [9]. Predicted values of spirometry, static lung volumes and IOS parameters were those of Global Lungs Initiative, European Community for Steel and Coal and of the KORA Study Group, respectively [10-12].

### 2.3 Statistical Analyses

Results are expressed as median [25^th^–75^th^ percentiles]. Univariate correlations were estimated using non parametric Spearman coefficients. A P value <0.05 was deemed significant. All statistical analyses were performed with Statview 5.0 software (SAS institute, Cary, North Carolina, USA).

Sample size calculation: due to its descriptive design our objective was to give observed percentages with a 95%CI range < 30% that can be obtained with a sample of 50 patients.

## 3 RESULTS

Patients’ description and lung function tests results are listed in Table **1**.

Based on FEV_1_% predicted, a moderate to moderately severe defect was observed, associated with mild hyperinflation (increase in RV).

IOS measurements (Rrs_5Hz_) were coherent with plethysmographic measurements of airway resistance (Raw), Rrs_5Hz_ being slightly higher. ΔRrs_5Hz-20Hz_ was increased in 28/50 patients (56%, 95%CI: 42-70, with a higher value than upper limit of normal) that suggested airway obstruction (SAOP diagnosis) before TLC measurement, while AX was elevated (above upper limit of normal) in 42/50 patients.

RV/TLC % predicted only correlated with FEV_1%_ predicted (rho= -0.528, p=0.0002) and FVC % predicted (rho= -0.531, p=0.0002), no significant correlation was evidenced with IOS parameters.

Fifteen patients (30%, 95%CI: 17-43) exhibited a significant FEV_1_ reversibility Fig. (**1**). Twenty-six patients (52%, 95%CI: 38-66) exhibited a significant IC reversibility Fig. (**1**), while 13 patients (26%, 95%CI: 14-38) exhibited a significant FVC reversibility.

Six patients were flow-responders (proximal response), 20 were volume-responders (increase in IC and/or FVC: distal response), 9 were flow and volume responders (overall response) and 15 patients were non responders. Overall, 35 / 50 (70%, 95%CI: 57-83) exhibited a significant bronchodilator response Fig. (**1**).

Table **2** describes the correlations between the different parameters assessing bronchodilator response. IOS parameters correlated to FEV_1_ response only, further suggesting their proximal assessment.

## DISCUSSION

Our descriptive prospective study demonstrates that the bronchodilator response of peripheral airways related to SAOP should be evaluated using the increase in IC rather than the conventional increase in FEV_1_ that assesses more central airways or even the increase in FVC. The study also shows that impedance measurements using the IOS method was not sufficiently sensitive to detect SAOP.

The first issue is whether the abnormal pattern of SAOP is always obstructive. From a physiological point of view (one compartment model for instance) [13] there are only three mechanisms explaining a decrease in FEV_1_, obstructive, restrictive and mixed defects. Since the pattern is not restrictive (normal TLC), the defect is obstructive. The associated hyperinflation (increase in RV and sometimes FRC) further suggests peripheral obstruction. Logically, the ATS/ERS consensus on clinical pulmonary function testing stated that this pattern would be related to patchy collapse of small airways early in exhalation after exclusion of technical problems. The discussion comes from the clinical conditions associated with SAOP such as restricted thoracic expansion (obesity, thoracic deformation without restrictive defect), justifying the label of “non-specific pattern” for some investigators [2, 14], which is indefensible from a physiological basis. Nevertheless, the SAOP is a syndrome related to non-specific conditions leading to peripheral obstruction. The present study shows that a significant proximal and/or distal reversibility was found in 70% of the patients that clearly demonstrates the obstructive nature of the defect.

The causes of SAOP were those previously described, COPD and asthma being the more prevalent [3]. The prevalence of 30% FEV_1_ reversibility is higher than that observed by Iyer *et al.* (13%, 95%CI: 11.2 to 14.8) [14], which may be related to recruitment bias. The more frequent reversibility, assessed on IC rather than FEV_1_, in peripheral airway diseases (volume response) has previously been reported in COPD [15].

One may wonder why a more prevalent IC response than FVC response was evidenced. Our hypothesis is that patients exhibiting SAOP are characterized by a specific tendency to collapse their small airways in exhalation (often remaining after bronchodilator assessment), even from FRC to RV explaining that slow inspiratory vital capacity is similarly reduced than expiratory FVC. Consequently, significant bronchodilation is more often obtained on FRC (and IC) than on RV (and FVC). Moreover, the measurement of FVC is obtained after deep inspiration that may reduce FVC due to its bronchoconstrictor effect [16]. Along this line, the recent study of Jarenback and colleagues, in COPD and control patients, showed that a significant correlation between Δ FVC% predicted and Δ RV% predicted after bronchodilation was only evidenced in the subgroup of patients with FEV_1_ < 65% predicted [17]. This result highlights that a reduction of hyperinflation is probably not necessarily associated with an increase in FVC.

Whether IOS parameters can assess peripheral airways obstruction remains debated because this statement mainly relies on modelling approaches [6, 18]. Based on simplistic physical grounds from healthy lungs, airway resistance measurement is deemed to assess proximal airways [1]. From previous studies, we suggested that 1) peripheral airways can significantly contribute to airway resistance in COPD patients based on a modelling approach [19], 2) airway resistance and specific airway resistance are often increased (~50%) in SAOP [3] and 3) some respiratory system resistance parameters could assess peripheral airways in COPD patients based on a statistical approach [20].

Despite this background, several arguments suggest that IOS parameters were not sensitive to detect small airways disease. Firstly, the IOS parameter ΔRrs_5Hz-20Hz_, deemed to assess peripheral obstruction, was inconsistently increased (56%) and its change after bronchodilator was associated with FEV_1_ change only. AX was more frequently elevated, but its increase does not necessarily traduce an obstructive defect, as demonstrated in interstitial lung diseases [21]. Since IOS does not rely on forced manoeuvres, it may reduce the effects of premature airway closure seen during forced spirometry manoeuvres, which could impede its ability to detect SAOP. Secondly, a significant increase in IC reflecting the decrease in dynamic hyperinflation, related to peripheral airways dilation, was not associated with any significant improvement in IOS parameters.

In conclusion, a significant reversibility is frequent (70%) in patients depicting a Small Airways Obstructive Pattern, mainly assessed by inspiratory capacity increase. Impulse oscillometry is of limited value in this context since only half of the patients were detected as obstructive using this method.

## Figures and Tables

**Fig. (1):**
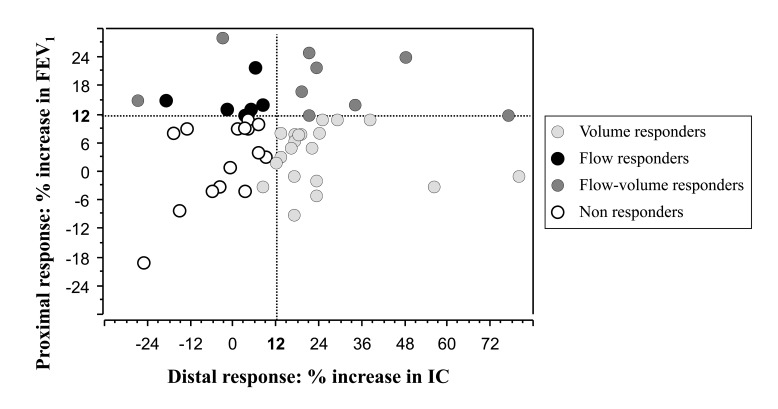
Relationship between proximal (increase in FEV_1_) and distal (increase in IC) response to bronchodilator. Dotted lines are 12% increase in FEV_1_ or IC as compared to baseline value. There was a non significant relationship between the two responses (p=0.465). Three patients, two with flow-responder group and one with non responder group, depicted a significant FVC response without significant IC response.

**Table 1:** Clinical and functional characteristics of the patients.

n or median [25^th^ – 75^th^ percentile]	
N patients	50
Sex ratio, F/M	27/23
Age, years	67 [50; 72]
BMI	26.9 [23.9; 32.4]
Underlying disease	–
asthma	15
COPD	26
bronchiectasis	6
sarcoidosis	2
interstitial disease	1
Tobacco history	–
current-smoker, n	9
ex-smoker, n	20
never-smoker, n	21
pack-years	33 [19; 50]
MRC score	2.0 [1.0; 3.0]
**Lung function**	–
FEV_1_, L	1.72 [1.27; 2.12]
FEV_1_, % predicted	59 [53; 65]
FEV_1_, Z-score	-2.53 [-2.96; -2.12]
FVC, L	2.22 [1.64; 2.79]
FVC, % predicted	63 [54; 67]
FVC, Z-score	-2.55 [-2.88; -2.20]
Slow inspiratory VC, L	2.22 [1.61; 2.80]
FEV_1_/FVC	0.74 [0.70; 0.78]
FEV_1_/FVC, % predicted	94 [90; 98]
FEV_1_/FVC, Z-score	-0.68 [-0.98; -0.18]
IC, L	1.62 [1.20; 2.20]
TLC, L	4.93 [4.16; 6.37]
TLC, % predicted	90 [85; 95]
FRC, L	3.31 [2.89; 4.28]
FRC, % predicted	110 [99; 131]
RV, L	2.80 [2.13; 3.40]
RV, % predicted	132 [117; 154]
RV/TLC	0.55 [0.49; 0.64]
RV/TLC, % predicted	142 [132; 152]
sRaw, kPa.s	1.63 [1.15; 2.26]
Raw, kPa.s/L	0.41 [0.30; 0.52]
*Impulse Oscillometry*	–
Zrs_5Hz_, kPa.s/L	0.48 [0.40; 0.63]
Zrs_5Hz_, % predicted	147 [128; 175]
Rrs_5Hz_, kPa.s/L	0.42 [0.34; 0.51]
Rrs_5Hz_, % predicted	134 [121; 167]
Xrs_5Hz_, kPa.s/L	-0.16 [-0.23; -0.11]
Xrs_5Hz_, % predicted	166 [106; 190]
∆Rrs_5Hz-20Hz_, kPa.s/L	0.15 [0.10; 0.21]
∆Rrs_5Hz-20Hz_, % predicted	213 [165; 296]
AX, Hz.kPa.s/L	1.41 [0.91; 1.97]
AX, % predicted	363 [268; 514]
*Bronchodilator response*	–
FEV_1_, % increase	+8 [+1; +12]
FVC, % increase	+6 [+1; +12]
IC, % increase	+12 [+3; +22]
Zrs_5Hz_, % decrease	0 [-9; +12]
Rrs_5Hz_, % decrease	+12 [-3; +21]
Xrs_5Hz_, (post-pre)	0.03 [0.01; 0.07]
∆Rrs_5Hz-20Hz_, % decrease	+25 [+10; +33]
AX, % decrease	+32 [+17; +47]

**Table 2:** Correlations between the bronchodilator response parameters.

Rho Values	FEV_1_, % Increase	FVC, % Increase	IC, % Increase	Zrs_5Hz_, % Decrease	Rrs_5Hz_, % Decrease	∆Rrs_5Hz-20Hz_, % Decrease	AX, % Decrease
FEV_1_, % increase							
FVC, % increase	**0.301**						
IC, % increase	0.084	**0.289**					
Zrs_5Hz_, % decrease	0.272	0.197	0.074				
Rrs_5Hz_, % decrease	0.269	0.164	0.025	**0.912**			
∆Rrs_5Hz-20Hz_, % decrease	**0.289**	0.161	0.013	**0.836**	**0.876**		
AX, % decrease	**0.295**	0.190	-0.032	**0.816**	**0.769**	**0.813**	
Xrs_5Hz_, (post-pre)	0.100	-0.001	0.039	0.243	**0.302**	**0.290**	**0.526**

Rho values are given for the correlations. Bold values denotes significant (p<0.05) correlations using Spearman test.

## LIST OF ABBREVIATIONS

SAOP: = Small Airways Obstructive Pattern
IOS: = Impulse Oscillometry
ΔRrs_5Hz-20Hz_: = Resistance from 5 Hz to 20 Hz
LLN: = Lower Limit of Normal
Zrs: = Impedance of the Respiratory System
Rrs: = Resistance of the Respiratory System
Xrs: = Reactance of the Respiratory System
AX: = Square Root of the Integrated Area of low Frequency Reactance
IC: = Inspiratory Capacity
